# Construction of chiral ZnO/BMP-2 dual functional coating on the surface of Ti implants for anti-infection and enhancement of osseointegration

**DOI:** 10.3389/fbioe.2026.1793619

**Published:** 2026-05-29

**Authors:** Yuan Fan, Haiyan Yao, Junchao Wei, Yipeng Hu

**Affiliations:** 1 Jiangxi Provincial Children’s Hospital, Nanchang, China; 2 School of Stomatology, Jiangxi Medical College, Nanchang University, Nanchang, China; 3 Jiangxi Provincial Key Laboratory of Oral Disease, Nanchang, China; 4 School of Chemistry and Chemical Engineering, Nanchang University, Nanchang, China

**Keywords:** antibacterial, chiral nanomaterials, osseointegration, Ti implant, ZnO

## Abstract

Titanium (Ti) implants are widely used in bone defect repair, however, postoperative infections and insufficient osseointegration would lead to implant failure. Therefore, it is much critical and necessary to construct antibacterial and osteogenic coatings on the surface of Ti implants to improve the successful rate of operation. Herein, D-penicillamine stabilized chiral ZnO nanoparticles (D-ZnO NPs) were prepared, which were loaded on the surface of Ti implants together with BMP-2 to construct a dual functional coating with antibacterial and osteogenic properties. The *in vitro* results demonstrated that the coating showed excellent antibacterial ability, and the antibacterial rates against *S. mutans*, *E. coli* and *S. aureus* were 86%, 98%, and 76%, respectively. Additionally, the coating could effectively induce macrophage polarization towards M2 phenotype, which modulated the local immune microenvironment. Furthermore, the introduction of bone morphogenetic protein-2 (BMP-2) promoted osteogenesis, which may enhance the osseointegration of Ti implants. This study provides a novel strategy to address the challenges of Ti implants, and offer the solid theoretical foundation for expanding the clinical applications of multifunctional Ti implants materials.

## 1 Introduction

Titanium (Ti) and its alloy have been widely used as implantable prostheses such as dental implants and joint replacement for restoring the morphology and function of hard tissues due to their excellent biocompatibility, mechanical properties and corrosion resistance (Wu et al., 2021; Che et al., 2024). However, bacterial infection and insufficient osseointegration are two challenges for Ti implants, which could reduce the long-term stability of implants and finally lead to the implant failure (Li et al., 2024; Yang et al., 2025; Zhou et al., 2024). It is reported that a mean prevalence of peri-implantitis is 22% and prevalence in which 5%–11% of dental implants failed (Zhang et al., 2021). Therefore, designing antibacterial and osteogenic coating on the surface of Ti implants is crucial for achieving ideal osseointegration (An et al., 2025).

Currently, antibacterial agents including antibiotics (Wang et al., 2024), quaternary ammonium salts (Jithin and Geetha, 2021), zinc oxide (Zhao et al., 2024), silver/copper (Ma et al., 2025) and their oxides have been loaded on the surface of Ti implants to kill peri-implant bacteria. Among these antibacterial agents, zinc oxide nanoparticles (ZnO NPs) have attracted much attention owing to their broad-spectrum antibacterial property, excellent biocompatibility and potential osteogenic ability (Chen et al., 2025; Lebaka et al., 2025; Nabil et al., 2025; Wang et al., 2026). However, ZnO NPs are easy to aggregate due to high surface energy, which limits its application in clinic. Therefore, many surface modification approaches have been proposed to improve biological properties of ZnO NPs (An et al., 2024). For example, ZnO NPs modified by chitosan showed stronger antibacterial ability than pure ZnO NPs (Yao et al., 2024). ZnO NPs modified with bovine serum albumin can significantly reduce their toxicity to human lung cancer cells and skin fibroblasts (Kelestemur et al., 2017). Furthermore, ZnO NPs prepare by L-cysteine showed excellent dispersion and better biosafety than ZnO NPs (Zhou et al., 2019). Chiral cysteine as inducer can be used to prepare chiral ZnO NPs, which showed excellent antiviral property and chiral dependence (Fan et al., 2023a). Therefore, chiral ZnO NPs prepared by chiral molecules may be much meaningful to expand the application of ZnO NPs, besides, it is also of great importance to further investigate the relationship between the structure and properties of nanomaterials.

Chiral molecules usually include the amino acids, protein, polysaccharides and DNA (Kim et al., 2025; Ding et al., 2025). The amino acids are small molecules with excellent biocompatibility, which are more easily grafted on the surface of nanomaterials compared with other biological macromolecules (Hao et al., 2025). Thus, small amino acids molecules are always used as ligand and surface capping agent to regulate the biological function of nanomaterials. Our group prepared chiral ZnO NPs stabilized with chiral arginine and found that chiral arginine on surface of ZnO NPs can enhance the interaction between bacteria and ZnO NPs, thereby improving effectively the antibacterial property (Fan et al., 2023b). Furthermore, D-arginine stabilized ZnO NPs showed stronger antibacterial property than that stabilized with L-arginine, showing obvious chiral dependence. Recently, our group found that D-penicillamine stabilized chiral ZnO NPs (D-ZnO NPs) exhibited better antibacterial ability than pure ZnO NPs (Yao et al., 2025). Furthermore, D-ZnO NPs can inhibit the inflammation reaction and promote the polarization of macrophages towards the M2 phenotype to improve immune microenvironment and finally promote the wound healing. Therefore, it is reasonable to hypothesize that Ti implants loaded with D-ZnO NPs may show excellent antibacterial property and immune regulation, which are beneficial for the osseointegration of Ti implants with surrounding bone tissue and provide a promising solution for peri-implantitis.

**FIGURE 1:**
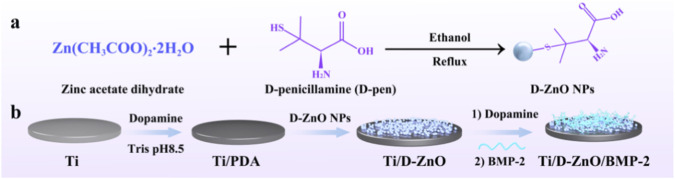
Schematic illustration of **(a)** preparation of D-ZnO NPs by sol-gel method and **(b)** D-ZnO/BMP-2 coating *via* layer by layer technique.

In this work, chiral D-ZnO NPs with penicillamine as ligand were prepared, and then assembled on the surface of Ti implant together with bone morphogenetic protein-2 (BMP-2) via layer by layer technique to construct dual functional coating (Ti/D-ZnO/BMP-2) with antibacterial and osteogenic properties (Figure 1). The *in vitro* results showed that the coatings exhibited excellent antibacterial activity for *S. mutans*, *E. coli* and *S. aureus,* and can promote the polarization of macrophages towards M2 phenotype, which provide good immune microenvironment for osseointegration. Furthermore, the introduction of BMP-2 can effectively induce cell differentiation into osteoblasts, thereby reinforcing the integration of Ti implants with the surrounding tissues. This work provide a promising strategy to address the challenges associated with bacterial infection and insufficient osseointegration for Ti implants, which show broad prospects in the field of orthopedic repair.

## 2 Materials and methods

### 2.1 Materials

Zinc acetate dihydrate (Zn(CH_3_COO)_2_•2H_2_O) and D-penicillamine were purchased from Sigma Aldrich. Potassium hydroxide (KOH) and Dimethyl sulfoxide (DMSO) were purchased from Sinopharm Chemical Reagent Co., Ltd (Shanghai, China). Ethanol was purchased from Xilong Technology Co., Ltd. Dopamine was purchased from Aladdin Scientific Co., Ltd (Shanghai, China). Bone morphogenetic protein-2 (BMP-2) was purchased from Genscript Biotechnology Co., Ltd (Nanjing, China). Round titanium sheets (diameter 10 mm, thickness 1 mm) were purchased from Xiu’ao Metal Ltd (Heibei, China).

Alizarin red S staining (ARS), 4% paraformaldehyde (PFA) and dulbecco’s modified eagle (DMEM) high glucose medium were purchased from Solarbio Co., Ltd (Beijing, China). The 3-(4,5-dimethylthiazol-2-yl)-2,5-diphenyltetrazolium bromide (MTT) assay kit, BCIP/NBT ALP Color Development Kit were purchased from Beyotime Co., Ltd (Shanghai, China). RIPA lysis buffer and BCA protein assay kit were purchased from Cwbio Biotechnology Co., Ltd (Taizhou, China). Living and dead bacterial staining kit was purchased from Uelandy Co., Ltd (Suzhou, China). DAPI staining solution was purchased from Shanghai Maokang Biotechnology Co., Ltd (Shanghai, China). ALP Detection Kit was purchased from Nanjing Jiancheng Biotechnology Co., Ltd (Nanjing, China).

### 2.2 Preparation of Ti/D-ZnO/BMP-2

Chiral D-ZnO NPs were synthesized by the sol-gel method according to reference (Yao et al., 2025). Briefly, 2.75 g of Zn(CH_3_COO)_2_•2H_2_O and 0.06 g of D-penicillamine were added to 90 mL of ethanol. The mixture was heated to 90 °C and refluxed for 1 h, then, 5 mL of KOH (1.7 M) ethanol solution was added. After 12 h, the white precipitates were centrifuged, washed, and lyophilized, and the obtained nanoparticles were denoted as D-ZnO NPs.

The coating of D-ZnO/BMP-2 was assembled on the surface of Ti implants *via* layer by layer technique. Briefly, 400 mg dopamine hydrochloride (DA) was dissolved in 200 mL tris-buffer solution (0.01 mol/L, pH 8.5), then Ti sheet was immersed in DA solution. Under the alkaline condition, DA polymerized on the surface of Ti sheet to form polydopamine (PDA). After 2 h, Ti sheet was removed and washed repeatedly by deionized water to obtain PDA modified Ti sheet (Ti/PDA). Then, Ti/PDA was immersed in 800 μg/mL D-ZnO NPs aqueous dispersion for 15 min, and washed with deionized water to remove the loosely absorbed particles and obtain Ti sheet loaded with D-ZnO NPs (Ti/D-ZnO). Subsequently, Ti/D-ZnO was soaked in DA solution for 10 min, washed, and then immersed in 5 μg/mL BMP-2 aqueous solution for 15 min, and then washed to finally obtain the Ti sheet with dual functional coating composed of D-ZnO NPs and BMP-2 (Ti/D-ZnO/BMP-2).

### 2.3 Characterization

The morphology and size were characterized by transmission electron microscopy (TEM, JEOL, JEM-2100, Japan). The elements of D-ZnO NPs were analyzed by X-ray photoelectron spectroscopy (XPS, Thermo Fisher Scientific, ESCALAB 250Xi, United States of America). The optical properties of D-ZnO NPs were investigated by ultraviolet-visible spectrometer (UV-Vis, JASCO, V-750, Japan) and circular dichroism (CD, JASCO, J-1500, Japan). The surface morphology of Ti, Ti/PDA, Ti/D-ZnO and Ti/D-ZnO/BMP-2 were observed by scanning electron microscopy (SEM, Thermo Fisher Scientific, Apero C HiVac, United States of America). The wettability of Ti, Ti/PDA, Ti/D-ZnO and Ti/D-ZnO/BMP-2 were measured by contact angle measuring instrument (KRÜSS, DSA100, Germany).

### 2.4 Biocompatibility assay

The biocompatibility of all samples was evaluated on mouse pre-osteoblast cells (MC3T3-E1) by MTT and the observation of cells morphology. In 24 wells, MC3T3-E1 cells at a density of 2 × 10^4^ cells/well were seeded on the surface of pure Ti sheet and Ti/D-ZnO/BMP-2, respectively, and incubated for 3 days. Then, the culture medium was removed and 1 mL MTT solution was added into 24 wells. After 4 h, the MTT solution was removed and 150 μL of DMSO was added into each well. Finally, the absorbance was measured using a microplate reader at 570 nm.

MC3T3-E1 cells at a density of 1 × 10^4^ cells/well were seeded on the surface of Ti sheet and Ti/D-ZnO/BMP-2 in 24 wells, respectively, and cultured for 3 days. The cells were fixed with 500 μL 4% PFA for 20 min. Then, the samples were washed by PBS and incubated with 0.5% TritonX-100 for 5 min. The cells were stained with TRITC-Phalloidin for 30 min and DAPI for 5–10s in the dark. The samples were washed with PBS and the cell morphology was observed by the fluorescence microscope (Leica, DMi8, Germany).

### 2.5 Antibacterial activity assay *in vitro*

*Streptococcus mutans* (*S. mutans*), *Escherichia coli* (*E. coli*) and *Staphylococcus aureus* (*S. aureus*) were used to evaluated the antibacterial activity of Ti/D-ZnO/BMP-2 by the plate counting method and live/dead staining. All three strains were incubated under the same conditions. Briefly, the Ti and Ti/D-ZnO/BMP-2 were placed in 24 wells and cultured with 500 μL of bacterial suspension (1 × 10^7^ CFU/mL)in a shaker at 37 °C, respectively. After 24 h, the samples were washed three times with PBS to remove the bacteria that had not adhered to the surface of coating. The cleaned samples were placed in the clean centrifuge tube. 1 mL PBS was added into the tube, and followed by ultrasonic and vibration treatment for three times to obtain bacteria that adhered to the surface of coatings. Then the bacterial suspension was serially diluted with PBS and 10 μL of each diluted sample was dropped onto LB (*E. coli* and *S. aureus*)/BHI(*S. mutans*) agar plates. After incubation in a shaker at 37 °C for 12 h, the bacterial colonies were counted and antibacterial rate was calculated with the following equation:

$$\text{Antibacterial rate }\left(\%\right)=\left(C_{0}-C\right)/C_{0}\times 100$$

where C_0_ is the number of bacterial colonies in the Ti group, and C is that of bacterial colonies in Ti/D-ZnO/BMP-2 group.

For live/dead staining assay, the Ti and Ti/D-ZnO/BMP-2 were placed in 24 wells and cultured with 500 μL of bacterial suspension (1 × 10^7^ CFU/mL) at 37 °C, respectively. After 24 h, the samples were washed by 0.85% NaCl solution. The bacteria were stained with DMAO/EthD-III (N, N-dimethylaniline N-oxide/Ethidium homodimer III) double staining kit for 15 min in the dark. The living bacteria was stained by DMAO (green fluorescence) and the dead bacteria was stained by EthD-III (red fluorescence). Then, the stained bacteria were observed by fluorescence microscopy (Leica, DMi8, Germany).

### 2.6 Macrophage polarization assay

Fluorescence staining was used to observe the morphology of macrophage on the surface of samples, and immunofluorescence staining were used to evaluate the expression of macrophage markers after sample treatment. RAW 264.7 cells at a density of 1 × 10^4^ cells/well were seeded on the surface of samples in 24 wells and incubated for 4 days. The cells were fixed with 4% PFA for 20 min and incubated with 0.5% TritonX-100 for 5 min. Then, the cells were stained with TRITC-Phalloidin for 30 min and DAPI for 5 s. The stained cells were observed by fluorescence microscopy.

Immunofluorescence staining: RAW 264.7 cells at a density of 5 × 10^4^ cells/well were seeded on the surface of samples in 48 wells and incubated. After 4 days, the cells were fixed, washed, blocked and incubated at 4 °C with anti-CD206 primary antibody. After 8 h, anti-iNOS antibody was added to incubate with cells at 4 °C for 8 h. Then, the cells were washed by PBST and incubated with the corresponding fluorescently labeled goat anti-mouse/rabbit secondary antibody for 8 h and DAPI for 5 min. The images were taken under the fluorescence microscope, and the fluorescent intensity was quantified and measured by ImageJ software.

### 2.7 Osteogenic activity assay

Alkaline phosphatase (ALP) staining and ALP activity: MC3T3-E1 cells at a density of 5 × 10^3^ cells/well incubated with Ti and Ti/D-ZnO/BMP-2, respectively. After 7 days, the cells were fixed, washed and stained by using the BCIP/NBT ALP Color Development Kit in the dark. After 18 h, the chromogenic reaction was terminated by washing with deionized water, and the dyeing results were captured by stereo microscope. ALP activity was analyzed with the ALP Detection Kit and BCA Protein Assay Kit according to the manufacturer’s instructions.

ARS staining: MC3T3-E1 cells were fixed with 4% PFA for 20 min after incubation with samples for 14 days. Subsequently, the samples were stained with 1% ARS for 2 h. Finally, deionized water was used to terminate further chromogenic reaction and stained cells were observed under a stereo microscope. According to the results of ARS staining, the ARS mean gray value were measured by ImageJ software.

Immunofluorescence staining of osteopontin (OPN) and osteocalcin (OCN): MC3T3-E1 cells were fixed, washed and blocked by 5% BSA for 1.5 h after incubation with samples. Then, the cells incubated with OPN/OCN antibody at 4 °C for 8 h, followed by incubation with goat anti-rabbit IgG fluorescence antibody at 4 °C for 8 h and DAPI for 15 min. Finally, the images were obtained by using fluorescence microscope.

### 2.8 Statistical analysis

All data were represented as mean ± standard deviation (SD). The significance analysis between the two groups was carried out by ANOVA and t-test. Statistical Significance was defined as *P < 0.05, **P < 0.01, ***P < 0.001.

## 3 Results and discussion

### 3.1 Characterization of Ti/D-ZnO/BMP-2

**FIGURE 2:**
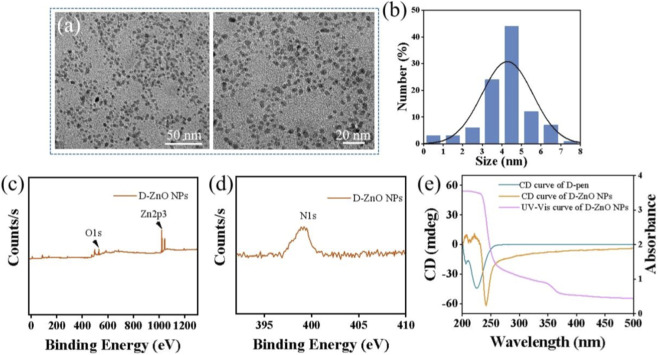
Characterization of D-ZnO NPs. **(a)** TEM images, **(b)** size distribution histograms, **(c)** XPS of O1s, **(d)** XPS of N1s, **(e)** CD and UV-Vis spectra of D-ZnO NPs.

D-ZnO NPs were prepared by sol-gel method, as shown in Figure 2a, the morphology of D-ZnO NPs was spherical nanoparticle with average size of 4.3 ± 1.3 nm (Figure 2b). XPS curve of D-ZnO NPs showed the characteristic signals of Zn2p3, O1s and N1s at 1,022, 529 and 399 eV (Figures 2c,d), respectively, which confirmed that the penicillamine was on the surface of ZnO NPs. UV-vis spectra and CD were used to investigate the optical properties of D-ZnO NPs. The results showed that the UV-vis characteristic absorption peak of D-ZnO NPs related to the exciton transition peak was around 350 nm. CD characteristic peak of D-pen was 225 nm, interestingly, that of D-ZnO NPs showed a red shift to 241 nm (Figure 2e), which was ascribed to the strong interaction between D-pen and ZnO, leading to the change of surface atoms arrangement. All results indicated that chiral ZnO NPs with D-pen as stabilizing ligand were prepared successfully.

**FIGURE 3:**
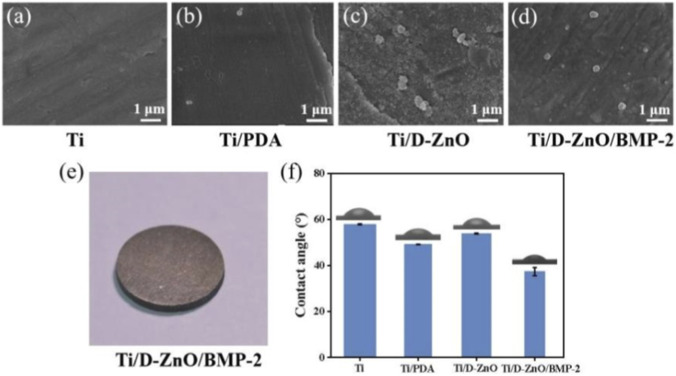
Characterization and physicochemical properties of Ti/D-ZnO/BMP-2. **(a)** SEM images of Ti, **(b)** Ti/PDA, **(c)** Ti/D-ZnO, and **(d)** Ti/D-ZnO/BMP-2; **(e)** Oblique view of Ti/D-ZnO/BMP-2, and **(f)** Water contact angle of Ti, Ti/PDA, Ti/D-ZnO and Ti/D-ZnO/BMP-2.

D-ZnO NPs and BMP-2 were assembled together on the surface of Ti sheets *via* layer-by-layer technique to construct dual functional coatings with antibacterial and osteogenic properties. SEM was used to observe the surface morphology of pure Ti, Ti/PDA, Ti/D-ZnO and Ti/D-ZnO/BMP-2. As shown in Figures 3a–d, the surface of pure Ti sheet was very smooth, while PDA layer formed on the surface of Ti sheet after treatment with dopamine solution, and some particles were found due to the aggregation of some PDA particles. After immersion in D-ZnO NPs suspensions, many nanoparticles were found on the surface of Ti/D-ZnO, which confirmed the successful loading of D-ZnO NPs on the surface of Ti sheet. However, the amount of D-ZnO NPs appearing on the surface of Ti/D-ZnO/BMP-2 was less than that of Ti/D-ZnO, which may be ascribed to the detachment of some D-ZnO NPs after washing. Furthermore, the black coating on the surface of the Ti sheet can be observed from the sample appearance in Figure 3e, confirming the successful construction of the coating. The surface roughness of Ti implants could affect the adhesion, proliferation, and differentiation of cells, thus affecting the effect of bone integration. SEM images showed that the surface roughness of samples was different. The surface roughness of pure Ti surface was very low, while that of Ti/PDA and Ti/D-ZnO groups were increased. Although the surface roughness of Ti/D-ZnO/BMP-2 group was lower than Ti/PDA and Ti/ZnO, it still presented rough topography. The results indicated that the surface roughness increased after the functionalization of Ti sheets, which may be favorable for cell adhesion and spreading.

The surface wettability is very vital for the properties of nanomaterials, and the hydrophilic properties is beneficial for the adherence and extension of cells on the surface of nanomaterials, which further induce the differentiation of osteoblasts to promote bone integration (Liu et al., 2025; Zhang et al., 2026). Thus, the water contact angle of all samples were investigated in this work. As shown in Figure 3f, the water contact angle of pure Ti plate was 57.8°, which decreased after dopamine treatment. After D-ZnO NPs treatment, the water contact angle increased from 49.1° to 53.8°, while that of Ti/D-ZnO/BMP-2 was 37.3°, which indicated that the surface coatings greatly improved the hydrophilicity of Ti sheet, which may have a positive contribution to the adhesion and proliferation of cells.

### 3.2 Biocompatibility of Ti/D-ZnO/BMP-2

**FIGURE 4:**
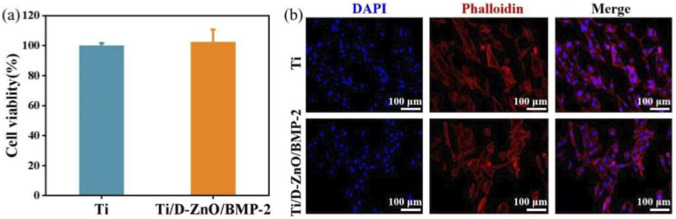
Biocompatibility of Ti/D-ZnO/BMP-2. **(a)** Cell viability of Ti and Ti/D-ZnO/BMP-2; **(b)** MC3T3-E1 cell morphology and adhesion on Ti and Ti/D-ZnO/BMP-2, respectively. All data were presented as mean ± SD (n = 3). *p < 0.05, **p < 0.01, and ***p < 0.001.

The biocompatibility is a crucial evaluation criteria to determine if the nanomaterials can be applied *in vivo* (Du et al., 2025). Cell viability assays is an effective method to evaluate biocompatibility of biological materials. In this work, MC3T3-E1 cells were incubated with Ti/D-ZnO/BMP-2, and the cell viability was tested by MTT. As shown in Figure 4a, Ti as biological materials show excellent biocomptibility that cell viability was 100%, while that of Ti/D-ZnO/BMP-2 group (102.3%) was better than Ti group, which demonstrated that the coating on the surface of Ti sheet possessed excellent biocompatibility. To further verify the biocompatibility of Ti/D-ZnO/BMP-2, fluorescence staining was used to observe the morphology of MC3T3-E1 cells on the surface of Ti sheets (Figure 4b). The cells were completely spread on the surface of Ti/D-ZnO/BMP-2 and presented the polygonal shape with filamentous pseudopodia, which was similar with cells in Ti group. All results demonstrated that Ti/D-ZnO/BMP-2 exhibited excellent biocompatibility, showing great potential for biomedical application.

### 3.3 Antibacterial activity of Ti/D-ZnO/BMP-2

**FIGURE 5:**
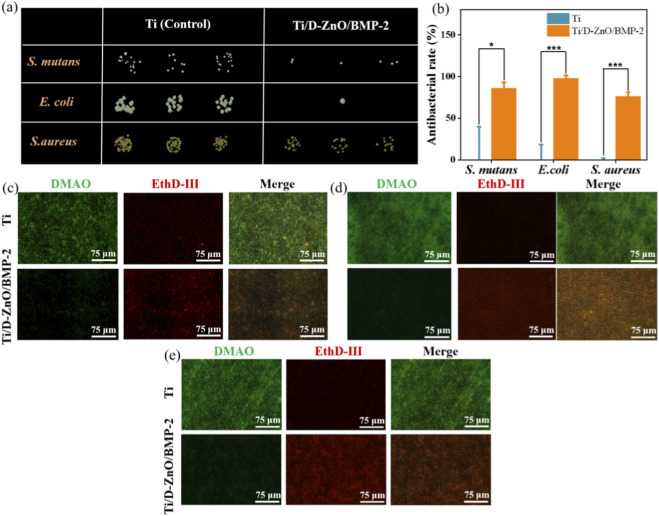
Antibacterial ability of Ti/D-ZnO/BMP-2. **(a)** Colonies photographs; **(b)** antibacterial rate against *S. mutans*, *E. coli* and *S. aureus,* respectively; **(c)** live/dead staining of *S. mutans*, **(d)** *E. coli* and **(e)** *S. aureus* after treated by Ti and Ti/D-ZnO/BMP-2, respectively. All data were presented as mean ± SD (n = 3). *p < 0.05, **p < 0.01, and ***p < 0.001.

In our previous work, our group confirmed that D-ZnO NPs showed excellent antibacterial property due to the strong interaction between bacteria and nanoparticles (Yao et al., 2025). Herein, the antibacterial ability of Ti and Ti/D-ZnO/BMP-2 were assessed with oral representative *S. mutans*, Gram-positive *S. aureus* and Gram-negative *E. coli*. As shown in Figure 5a, compared with Ti (control) group, the number of bacterial colonies of Ti/D-ZnO/BMP-2 group were much fewer. The antibacterial rate against *S. mutans*, *S. aureus* and *E. coli* was 86%, 98%, and 76% after Ti/D-ZnO/BMP-2 treatment, respectively (Figure 5b). These results implied that Ti/D-ZnO/BMP-2 displayed good antibacterial activity, which was ascribed to the excellent antibacterial effect of D-ZnO NPs.

Living/dead bacterial staining was used to further evaluate the antibacterial ability of Ti/D-ZnO/BMP-2. When Ti sheets were cultured with *S. mutans*, the red fluorescence was weak, which indicated that Ti group showed weak antibacterial effect for *S. mutans* (Figure 5c). While the strong red fluorescence was observed on the surface of Ti/D-ZnO/BMP-2, which showed extensive bacteria death, indicating the good antibacterial effect of dual functional coatings for *S. mutans.* Furthermore, when Ti sheets were cultured with *E. coli* and *S. aureus*, respectively, the results showed almost no bacterial death, which confirmed that Ti sheets showed almost no antibacterial effect on these two bacteria (Figures 5d,e). In the Ti/D-ZnO/BMP-2 group, high intensity red fluorescence appeared, indicating a large number of bacterial death, which further proved that the D-ZnO/BMP-2 coating on the Ti surface displayed excellent antibacterial performance.

### 3.4 Macrophage polarization effect of Ti/D-ZnO/BMP-2

**FIGURE 6:**
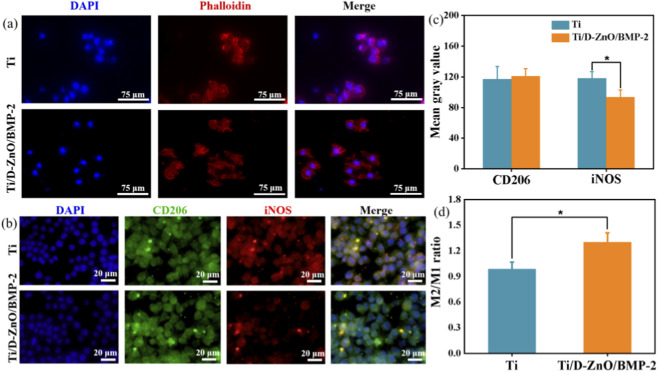
Effect of Ti/D-ZnO/BMP-2 on macrophages polarization. **(a)** Fluorescent staining images of macrophages; **(b)** immunofluorescence staining images of CD206 and iNOS after macrophages cultured with Ti and Ti/D-ZnO/BMP-2 for 4 days, respectively; **(c)** mean fluorescence intensity of CD206 and iNOS; **(d)** the ratio of mean fluorescence intensity of CD206 to that of iNOS (M2/M1 ration). All data were presented as mean ± SD (n = 3). *p < 0.05, **p < 0.01, and ***p < 0.001.

During the bone repair process after Ti implantation, a favorable immune microenvironment plays a crucial role in enhanced osseointegration (Xiong et al., 2022). Macrophages are main immune cells and its’ polarization phenotype determine the state of the immune microenvironment of tissue surrounding Ti implants (Qiao et al., 2023). Generally, macrophages can differentiate into M1 and M2 phenotypes. M1-type with round shape secretes inflammatory factors such as TNF-α and IL-1β, which can trigger the process of bone resorption, while M2-type with elongated shape secretes antiinflammatory factors such as IL-10 and TGF-β, which can promote bone repair (Wei et al., 2025). Therefore, phenotypes of macrophages in the microenvironment surrounding Ti implants is very crucial for osseointegration. Many investigations have shown that bioactive materials such as BMP-2, VEGF, IL-4, metal ions (Zn^+^, Mg^2+^) and phytoconstituents (Rahaman and Mukherjee, 2026), can be designed into the coatings on the surface of Ti implants to induce the polarization of macrophages towards M2 type, which can trigger the remodeling of immune microenvironment, induce bone regeneration, and ultimately achieve good osseointegration. In this work, the effect of Ti/D-ZnO/BMP-2 on macrophages polarization was investigated. The fluorescence staining was used to observe the morphology of macrophages on the surface of Ti and Ti/D-ZnO/BMP-2, respectively. As shown in Figure 6a, the RAW264.7 cells in Ti group showed the round morphology and few cells displayed filamentous pseudopods, which indicated that Ti implants may not significantly induce excessive polarization of RAW264.7 cells and could reduce macrophage immune responses. In Ti/D-ZnO/BMP-2 group, macrophages showed the uniform and fully extended state with obvious pseudopodia, which demonstrated that macrophages could adhere and stretch well on the surface of Ti/D-ZnO/BMP-2, and the phenotypes of macrophages may change.

The effect of Ti/D-ZnO/BMP-2 on macrophages polarization was evaluated by immunofluorescence staining. The iNOS of M1 specific marker and CD206 of M2 specific marker were used to characterize the phenotype of macrophages. As shown in Figure 6b, the expression level of CD206 in the Ti/D-ZnO/BMP-2 group was significantly higher than that in the Ti group, which implied that Ti/D-ZnO/BMP-2 can promote more macrophages to polarize towards M2 type. Meanwhile, the results showed that the expression level of iNOS in the Ti group was higher than that in the Ti/D-ZnO/BMP-2 group, indicating that more macrophages in the Ti group could polarize towards M1 type. The quantitative results of average fluorescence intensity (Figure 6c) also showed that the expression level of CD206 was higher in the Ti/D-ZnO/BMP-2 group, while the expression level of iNOS was lower than that in Ti group. The ratio of M2 to M1 macrophages in the Ti and Ti/D-ZnO/BMP-2 groups was 0.99 and 1.30, respectively (Figure 6d). All results demonstrated that the dual functional coating could induce macrophage polarization towards M2 type, providing the favorable immune microenvironment for the integration of Ti implants with surrounding bone tissue, thus promoting osseointegration.

### 3.5 Osteogenic ability of Ti/D-ZnO/BMP-2

**FIGURE 7:**
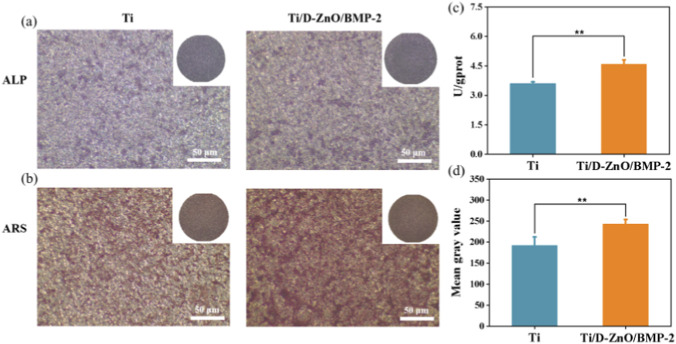
Osteogenic property of Ti/D-ZnO/BMP-2 Ti/D-ZnO/BMP-2. **(a)** ALP and **(b)** ARS staining images of MC3T3-E1 cultured with Ti and Ti/D-ZnO/BMP-2 for 7 and 14 days, respectively; **(c)** ALP quantitative analysis and **(d)** ARS mean gray value of Ti and Ti/D-ZnO/BMP-2. All data were presented as mean ± SD (n = 3). *p < 0.05, **p < 0.01, and ***p < 0.001.

BMP-2 is an important bone inducing growth factor that can promote the differentiation of MC3T3-E1 cells into osteoblasts (Lou et al., 2025). The introduction of BMP-2 into surface coating contribute to promoting osteogenesis and enhancing the osseointegration of Ti implant with surrounding bone tissue. The cells incubated with Ti and Ti/D-ZnO/BMP-2 for 7 days to detect the early osteogenic marker ALP, respectively. As shown in Figure 7a, the results showed that the expression level of ALP in the Ti/D-ZnO/BMP-2 group was 4.6 U/gprot, which was higher than that in the Ti group (3.6 U/gprot), indicating that Ti/D-ZnO/BMP-2 exhibited good osteogenic properties. ARS staining can be used to evaluate its osteogenic performance in late osteogenesis *via* the observation of calcium salt deposition. The ARS staining results showed that the Ti/D-ZnO/BMP-2 group had more calcium salt deposition on the surface than the Ti group (Figure 7b), which may be ascribed to that Ti/D-ZnO/BMP-2 promoted more MC3T3-E1 cells to differentiate into osteoblasts. In addition, the results of ALP quantification experiment and ARS mean gray value were consistent with the above staining results (Figures 7c,d), indicating that Ti/D-ZnO/BMP-2 displayed excellent osteogenic properties.

**FIGURE 8:**
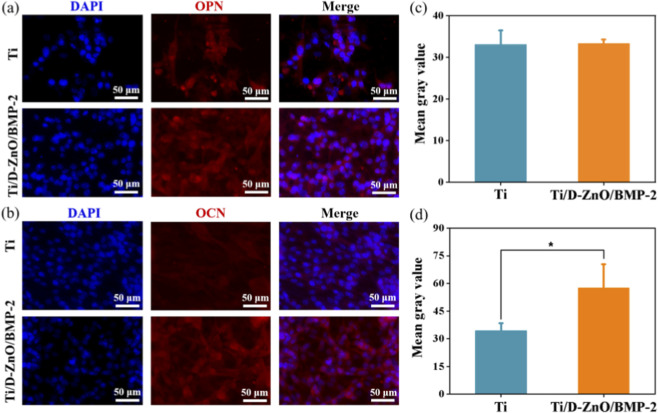
Osteogenic property of Ti/D-ZnO/BMP-2. **(a)** Immunofluorescence staining images of OPN and **(b)** OCN after MC3T3-E1 cells cultured with Ti and Ti/D-ZnO/BMP-2 for 7 days, respectively; **(c)** Quantitative analysis of OPN and **(d)** OCN. All data were presented as mean ± SD (n = 3). *p < 0.05, **p < 0.01, and ***p < 0.001.

During the process of bone remodeling, extracellular matrix proteins such as OPN and OCN are actively expressed. The expression levels of OPN and OCN during osteogenesis were detected using immunofluorescence. As shown in Figure 8, the expression levels of OPN in the extracellular matrix on the surfaces of Ti and Ti/D-ZnO/BMP-2 were not significantly different, while the expression levels of OCN were significantly different. The OCN expression level in the Ti/D-ZnO/BMP-2 group was higher than that in the Ti group, which further confirmed that Ti/D-ZnO/BMP-2 showed good osteogenic properties and can effectively promote the integration of implants with surrounding bone tissue.

## 4 Conclusion

In this work, a dual functional coating with antibacterial and osteogenic properties was constructed on the surface of Ti implants by layer by layer technique. Ti/D-ZnO/BMP-2 showed excellent biocompatibility and antibacterial activity against *S. mutans*, *E. coli* and *S. aureus.* Furthermore, the coating could promote the polarization of macrophages towards M2, greatly improve the expression of CD206 and inhibit that of iNOS, which could improve local immune microenvironment, thus creating the favorable conditions for bone integration. Meanwhile, the introduction of BMP-2 enhanced the osteogenic induction ability of the coating, and the improvement of ALP activity, calcium salt deposition and OCN expression all confirmed the osteogenic ability of the coating. In summary, Ti/D-ZnO/BMP-2 showed excellent antibacterial and osteogenic effects, providing a promising strategy for solving the problems of Ti implant infection and insufficient bone integration.

## Data Availability

The original contributions presented in the study are included in the article/supplementary material, further inquiries can be directed to the corresponding authors.
